# Comparison of psychedelic and near-death or other non-ordinary experiences in changing attitudes about death and dying

**DOI:** 10.1371/journal.pone.0271926

**Published:** 2022-08-24

**Authors:** Mary M. Sweeney, Sandeep Nayak, Ethan S. Hurwitz, Lisa N. Mitchell, T. Cody Swift, Roland R. Griffiths

**Affiliations:** 1 Department of Psychiatry and Behavioral Sciences, Behavioral Pharmacology Research Unit, Center for Psychedelics and Consciousness Research, Johns Hopkins University School of Medicine, Baltimore, Maryland, United States of America; 2 Department of Neurosciences, Johns Hopkins University School of Medicine, Baltimore, Maryland, United States of America; Virginia Commonwealth University, UNITED STATES

## Abstract

Both psychedelic drug experiences and near-death experiences can occasion changes in perspectives on death and dying, but there have been few direct comparisons of these phenomena. This study directly compared psychedelic occasioned and non-drug experiences which altered individuals’ beliefs about death. Individuals who reported an experience that altered their beliefs about death occasioned by either a psychedelic drug or a near-death or other non-ordinary experience completed an online survey. Circumstances of the experience, mystical and near-death subjective features, changes in attitudes about death, and other persisting effects were evaluated. The study sample (*n* = 3192) included five groups: non-drug near-death or other non-ordinary experiences (*n* = 933), and drug experiences occasioned by lysergic acid diethylamide (LSD) (*n* = 904), psilocybin (*n* = 766), ayahuasca (*n* = 282), or *N*,*N-*dimethyltryptamine (DMT) (*n* = 307). Analyses of differences in experiences were adjusted statistically for demographic differences between groups. Compared to the psychedelic groups, the non-drug group was more likely to report being unconscious, clinically dead, and that their life was in imminent danger. The groups were remarkably similar in the reported changes in death attitudes attributed to the experience, including a reduced fear of death and high ratings of positive persisting effects and personal meaning, spiritual significance, and psychological insight. Although both psychedelic and non-drug participants showed robust increases on standardized measures of mystical and near-death experiences, these measures were significantly greater in the psychedelic participants. Non-drug participants were more likely to rate their experiences as the single most meaningful of their lives. Comparing across psychedelic substances, ayahuasca and DMT groups tended report stronger and more positive enduring consequences of the experience than the psilocybin and LSD groups, which were largely indistinguishable. These data provide a detailed characterization and comparison of psychedelic occasioned and non-drug experiences that changed attitudes about death and suggest the importance of future prospective psychedelic administration studies.

## Introduction

Classic psychedelic drugs including psilocybin, lysergic acid diethylamide (LSD), and *N*,*N-*dimethyltryptamine (DMT) produce effects ranging from mild changes in sensory perception to mystical-type experiences characterized by an authoritative sense of unity or oneness and sacredness, deep positive mood, ineffability, and transcendence of time and space [1, 2]. The range of effects occasioned by psychedelic drugs is dependent on dose, personal and biological characteristics of the person ingesting the drug, and the setting in which the drug is ingested [3–5]. Prior double-blind research examining the effects of classic psychedelics has shown that drug-occasioned mystical experiences are associated with positive changes in attitudes, mood and behavior that persist months to more than a year after drug administration [6–9]. The subjective effects of psychedelic drugs are attributable to neuropharmacological action as agonists at the serotonin 2A (5-HT2A) receptor subtype [10, 11]. It is hypothesized that 5-HT2A receptor agonism results in increased neural plasticity, changes in brain network connectivity, and possibly relaxation of high-level beliefs, which may underlie the acute transcendent mystical-type psychedelic experience and its persisting long-term effects [10, 12]. The subjective features of psychedelic experiences have been a primary driver of interest in therapeutic application to mood disorders and other psychiatric conditions [13].

One commonly reported consequence of psychedelic experiences is a fundamental change in perspective on death and dying. Following exposure to psilocybin under double-blind conditions, one research participant described, “The sense that all is One, that I experienced the essence of the universe and the knowing that God asks nothing of us except to receive love. I am not alone. I do not fear death,” [14, 15]. Transformative effects of psychedelics on beliefs about death is consistent with data from recent research in which classic psychedelics have demonstrated efficacy for anxiety reduction among patients with life-threatening diseases [16–20]. For example, in a randomized trial among 51 cancer patients with clinically significant anxiety or depressive symptoms, high-dose psilocybin (22 or 30 mg/70 kg) delivered in structured therapeutic environment resulted in significant increases in ratings of death acceptance and death transcendence, as well as decreases in anxiety about death [17]. Although these data are encouraging for clinical application, few studies have examined the effects of psychedelic experiences on attitudes about death occurring outside of the clinical context of end-of-life anxiety. Recent online cross-sectional survey data evaluating thousands of retrospective reports of psychedelic experiences occurring in naturalistic settings have provided some relevant data. A survey examining naturalistic psychedelic experiences described as “personal encounters with God” found that most participants (67–77% across different psychedelic drug groups) reported a decrease in their fear of death resulting from their experience [21]. In a survey evaluating DMT-occasioned encounters with “a seemingly autonomous entity” (e.g., being, guide, spirit, alien), 76% of respondents reported a positive desirable change in their attitudes about death [22]. Although these “encounter” experiences represent only small subset of experiences occasioned by psychedelic drugs, the results suggest the value for further studies to precisely characterize changes in death attitudes following use of psychedelics.

Acute, time-limited experiences that fundamentally alter perspectives of death and dying can also occur spontaneously in the absence of any drug, as with near-death experiences or related phenomena such as out-of-body experiences [23–27]. Greyson and colleagues developed a quantitative, 16-item scale to assess near-death experiences based on empirical evaluation of 80 elements of near-death experiences drawn from published phenomenological accounts of such experiences [28]. Prominent features of the near-death experience assessed by the Greyson Near-Death Experience Scale include feelings of altered time perception, seeing scenes from the past or the future, joy and peace, unity with the world, feeling separated from the physical body, and seeming to encounter mystical beings, deceased spirits, or religious figures [28]. Like psychedelic experiences, near-death experiences can be characterized using the mystical experience framework and are reported to result in persisting positive changes in attitudes and behaviors such as increased spiritual practices and improved self-esteem [29–33]. Near-death experiences are generally understood to result in decreases in fear of death, though limited quantitative data are currently available [34]. When compared on a standardized metric, those who have had near-death experiences tend to have less death anxiety relative to control participants who have not had near-death experiences [29, 35]. For example, recent online survey data showed that relative to control participants (*n* = 104), participants who had a near-death experience (*n* = 102) tended to have lower ratings for items assessing fear of death and higher ratings for so-called death “approach acceptance”, which assesses belief in a happy afterlife [29]. The acute, time-limited, and mystical characteristics of near-death experiences along with their enduring effects, including changes in death attitudes, suggests value in comparing shared phenomenological features of near-death experiences with those occasioned by psychedelic drugs [36].

There have been few direct comparisons of psychedelic experiences with near-death experiences on similar quantitative metrics. A comparison of narrative reports of near-death experiences and psychedelic experiences using natural language processing suggests some shared semantic content, particularly the use of terms related to the visual components of the experience (e.g., “color”, “visual”, “pattern”, “saw”) [37]. In addition, placebo-controlled laboratory administration of DMT resulted in increased ratings of near-death experience features on the Greyson Near-Death Experience Scale [38]. A recent study using a mail-in questionnaire to directly compare ratings from persons with near-death experiences (*n* = 161) and psychedelic drug-occasioned experiences (*n* = 51) on a common metric suggest similar subjective ratings for some aspects of the experience including changes in time perception, unusual sensations, and sudden understanding, but generally lower ratings in the drug group for some aspects of the experience including feelings of coming close to a border or point of no return, encountering a presence or entity, and feelings of dying or being dead [39]. These direct comparisons were somewhat limited by the small sample of persons with drug-occasioned experiences who completed the questionnaire, which were predominantly occasioned by psilocybin or LSD [39]. Different psychedelic drugs may differ in the extent to which they resemble near-death experiences either due to pharmacological differences, route of administration, or setting. Although psilocybin, LSD, and DMT are all agonists at the 5-HT2A receptor, they may differ in binding affinity at 5-HT2A, other 5-HT receptors, or at receptors for other neurotransmitters such as dopamine or glutamate, potentially contributing to differences in subjective effects [40]. Further, naturalistic experiences with different drugs vary systematically with respect to route of administration and setting. For example, psilocybin and LSD are most often taken orally, DMT is commonly administered via inhalation or ingested orally from ayahuasca brew in a ceremonial context. These differences in route of administration and setting may affect both the intensity of the subjective effects during the experience or the interpretation of the meaning of the experience. Controlling for other differences between drug groups, such as demographic differences, would also be important when evaluating and comparing psychedelic experiences occasioned by different drugs and near-death experiences.

Taken together, prior research suggests that both psychedelic drugs and non-drug near-death experiences or related phenomena such as out-of-body experiences decrease fear of death; however greater understanding of changes in death attitudes resulting from such experiences is warranted. It is worthwhile to compare drug-occasioned experiences that transformed perspectives on death and dying with non-drug occasioned experiences to understand shared phenomenological characteristics and possible similarities and differences in enduring effects. This information will be useful in characterizing experiences that change perspectives on death and dying and may inform clinical practice in reducing end-of-life anxiety and distress. Thus, the purpose of the present study was to directly compare psychedelic experiences and non-drug experiences that were reported to change perspectives on death and dying. Using an online survey, we recruited persons who reported changed beliefs about death that they attributed: 1. to an experience occasioned by psilocybin, LSD, ayahuasca, or DMT, or 2. to a near-death or other non-ordinary experience they had in absence of using such substances. The study assessed the phenomenological features of the experience, accompanying changes in death attitudes, persisting effects, and ratings of the experience relative to other life events on common standardized metrics.

## Materials and methods

### Participant recruitment

Participants were recruited via internet advertisements, email invitations, organizational newsletters, blog postings, and online social networks, particularly those relevant to either near-death experiences (e.g., near-death experience blogs; iands.org) or psychedelic drugs (e.g., bluelight.org; erowid.org). Two versions of the survey were created, one intended for near-death experiences (but inclusive of other non-ordinary experiences such as out-of-body experiences) and another for psychedelic experiences. Online advertisements contained a link to the appropriate survey. Both surveys were administered using Qualtrics survey design and hosting platform (www.qualtrics.com). Written consent was obtained using the first page of the survey on which the participant was instructed that by selecting “Begin survey,” they were affirming they had read the study description, they fulfilled the eligibility criteria, and they voluntarily agreed to participate. On the consent page for both surveys, the purpose of the study was described as “to characterize an experience that fundamentally altered your beliefs or understanding about death and dying." The Psychedelic Group completed the survey based on an experience that occurred after taking a classic psychedelic including psilocybin, LSD, ayahuasca, or DMT other than ayahuasca (e.g., smoked/vaporized N,N-DMT; but not 5-MeO-DMT) (hereafter referred to as DMT). The Non-Drug Group completed the survey based on a “near-death or other non-ordinary experience”. Consent stated that participation was anonymous, the respondent could exit the survey at any time, and any responses would only be used if the respondent completed the survey. No compensation was provided. In the Non-Drug Group survey, brief videos were used to introduce the survey (approximately 1 min) and interspersed videos (all less than 30 sec) between sections to increase engagement and encourage completion. The Institutional Review Board of the Johns Hopkins University School of Medicine approved all study procedures.

### Inclusion/Exclusion criteria

To qualify to take the survey, participants must have indicated being: 1. 18 years of age or older; 2. able to read, write, and speak English fluently; 3. reported not taking the survey previously; 4. reported having “an experience that fundamentally altered your beliefs or understanding about death and dying.” Individuals taking the psychedelic survey also indicated that 5. the experience was a result of taking a classic psychedelic. Participants who met the inclusion criteria were presented the remaining survey items. Participants were instructed to answer the survey items in response to their single most memorable experience. Exclusion criteria included: 1. indicating their response should be excluded from analysis (based on an end-of-survey question); 2. concurrent non-medical use of any other psychoactive drug (except caffeine or nicotine), including use of more than one type of classic psychedelic substance among psychedelic survey respondents; and 3. answering the survey based on multiple experiences rather than a single experience.

### Survey measures

The survey assessed the content and context of participants’ experiences using multiple-choice and open-ended response items and determined basic demographics. Participants in the Psychedelic Group indicated which of several classic psychedelics they believe they had taken using multiple-choice (i.e., by selecting one of several mutually exclusive options). Participants answered multiple choice items to describe the duration of the experience and whether their life was in danger. Open-ended response items included a description of the experience and the context in which it occurred.

Participants completed assessments to describe their experience and its enduring effects, including the 16-item Greyson Near-Death Experience (NDE) Scale, which includes subscales to quantify the *cognitive*, *affective*, *paranormal*, and *transcendental* aspects of NDEs [28]. *Cognitive* subscale items assessed to what extent time and thoughts were speeded up, whether scenes from the past came back to the respondents, and whether they suddenly seemed to understand “everything”. The *affective* subscale assessed feelings of peace/joy/unity and the perception of a brilliant light. The *paranormal* subscale assessed whether senses were more vivid than usual, whether they seemed to be aware of things as if by extrasensory perception, experienced scenes from the future, and felt separated from the physical body. The *transcendental* subscale assessed whether they seemed to enter an unearthly world, encountered a mystical being or presence, saw deceased spirits, or encountered a border or point of no return.

The subjective characteristics of the acute experience and overall life changes attributed to the experience were assessed with the Hallucinogen Rating Scale (HRS) [41], selected items from the States of Consciousness Questionnaire [42], the Persisting Effects Questionnaire [42], and the Mystical Experience Questionnaire (MEQ30) [1, 43]. A single questionnaire item asked "During your experience, did you encounter something that someone might call "God" (e.g., the God of your understanding), with response option being yes or no. The MEQ30 included subscales to quantify the *mystical* (including feelings of unity, sacredness, and noetic quality; direct knowledge or insight), *positive mood* (e.g., awe, joy), *transcendence of time and space*, and *ineffability* aspects of mystical experience. A “complete” mystical experience according to the MEQ30 was defined *a priori* as having scores 60% or above on all four subscales [1].

Participants responded to items on the Revised Death Attitude Profile (DAP-R) [44] and indicated their views before and after the experience. The DAP-R includes subscales for *fear of death* (e.g., “The prospects of my own death arouse anxiety in me.”), *death avoidance* (e.g., “I avoid death thoughts at all costs.”), *neutral acceptance* (e.g., “I would neither fear death nor welcome it.”), *approach acceptance* (e.g., “Death is an entrance to a place of ultimate satisfaction.”), and *escape acceptance* (e.g., “Death provides an escape from this terrible world.”). Items were rated on a 7-point Likert scale from disagree strongly to agree strongly. Differences in death attitudes on the DAP-R subscales before and after the experience were calculated as change scores, with negative values indicating a decrease in the score on that subscale after the experience relative to before the experience.

### Statistical analyses

Descriptive statistics for demographic variables and survey measures are reported for the Non-Drug Group and the Psychedelic Group, as well as separately for each drug group (psilocybin, LSD, ayahuasca, and DMT other than ayahuasca). Comparisons between the Non-Drug Group and the Psychedelic Group were made using linear regressions for continuous outcomes (e.g., proportion of total scores and subscales on standardized questionnaires such as the MEQ) and logistic regressions for dichotomous outcomes (e.g., percentage of participants meeting criteria for a complete mystical experience on the MEQ) with group as a factor, while controlling for demographic variables of age at the time of survey (continuous), sex (male or female), race and ethnicity (white and not Hispanic or other race/ethnicity), residing in the United States (US or other countries), ever married or living with partner (yes or no), annual income (≥ 50K USD or <50K USD), and education (with or without an undergraduate degree). Standardized β are reported for linear regressions and odds ratios (OR) are reported for logistic regressions as indices of effect size to enable comparisons between groups on a common metric across outcomes. Standardized β can be interpreted as the standardized mean difference between groups after adjusting for covariates—similar to an "adjusted Cohen’s d". Odds ratios can be interpreted as the odds of the binary outcome in the psychedelic group divided by the odds of the outcome in the non-psychedelic group, after adjusting for covariates. Pearson correlations between MEQ and NDE Scale scores were examined for the total sample and within the Non-Drug and Psychedelic Group, similar to prior research [38].

Comparisons between the drug groups (i.e., Psilocybin, LSD, Ayahuasca, DMT, and Non-Drug) were conducted using generalized linear models with a logit link and Type III Sums of Squares, including the seven covariates described above. Continuous data were analyzed using ANOVA with the same covariates and Type III Sums of Squares. For both continuous and categorical analyses, pairwise comparisons among the drug groups were adjusted using the Bonferroni method to control for Type I error rate.

Results for all the analyses described above, including those which also used a Bonferroni correction, were considered significant when p≤0.001. These conservative statistical criteria were adopted from prior research and used to focus on robust differences between groups and account for multiple comparisons [21]. Because large sample sizes can detect statistically significant differences that represent minor practical differences, discussion of the results will focus variables that are significantly different between groups AND for which the difference is >10%.

## Results

### Survey completion and final sample

During recruitment (12/04/2015-04/05/2018), 15,956 respondents began the survey. Of these, 1,510 did not meet eligibility criteria and 1,123 quit the survey before eligibility could be determined. Of those who met eligibility, 8,157 did not complete the survey and 55 completed the survey but indicated their data should not be used according to an end-of-survey response. Responses from the psychedelic survey must have endorsed use of psilocybin or psilocybin mushrooms, LSD, ayahuasca, or DMT (but not 5-MeO-DMT) to be included in the analysis (*n* = 552 excluded). Responses were also excluded if the participant indicated concurrent alcohol use (*n* = 202), concurrent cannabis use (*n* = 914), concurrent non-medical use of any other psychoactive drug except caffeine, nicotine, or use of more than one classic psychedelic among psychedelic survey respondents (*n* = 133), answered the survey based on multiple experiences rather than a single experience (*n* = 111), or provided non-English or otherwise uninterpretable text responses (*n* = 7). The final sample included 3,192 responses, 2,259 in the Psychedelic Group and 933 in the Non-Drug Group. Among the Psychedelic Group, 40% indicated their experience was occasioned by LSD (*n* = 904), 34% indicated psilocybin or psilocybin mushrooms (*n* = 766), 12% indicated ayahuasca (*n* = 282), and 14% indicated smoked/vaporized DMT other than ayahuasca (*n* = 307). The median time to complete the survey was 45 minutes.

### Participant characteristics

Table 1 presents demographic information for both the Psychedelic Group and the Non-Drug Group. Participants in the Non-Drug Group tended to be older on average than those in the Psychedelic group, both at the time of the experience (mean age 32.0 vs. 25.0 years) and at the time of study participation (mean age 55.2 vs. 31.7 years). The sample was predominantly White and not Hispanic. There were fewer females in the Psychedelic Group (22%) relative to the Non-Drug Group (68%). The Psychedelic Group had fewer participants who had achieved a bachelor’s degree or higher in education (43% vs. 54%), more participants who had never been married (54% vs. 16%), and fewer earning above $50K per year (47% vs. 58%) relative to the Non-Drug Group. Fewer participants in the Psychedelic Group reported living in the United States (61% vs. 69%).

**Table 1:** Participant characteristics in the Non-Drug Group and combined Psychedelic Group.

	Non-Drug	Psychedelic	
	*n* = 933	*n* = 2259	*p* value^1^^,^^2^
Mean age at study participation in years (SD)	55.2 (13.5)	31.7 (13.5)	≤.001
Mean age at time of experience in years (SD)	32.0 (16.4)	25.0 (9.5)	≤.001
Sex (%)			≤.001
Male	32%	78%	
Female	68%	22%	
Race (%)			ns^3^
White	89%	84%	
More than one race	6%	10%	
Asian	2%	4%	
Native American	1%	2%	
African American/Black	2%	<1%	
Native Hawaiian or Pacific Islander	<1%	<1%	
Ethnicity (% Hispanic)	7%	10%	≤.001
Education (%)			≤.001
Less than a Bachelor’s Degree	46%	57%	
Bachelor’s Degree or higher	54%	43%	
Annual household income (%)			≤.001
Less than $50,000	42%	53%	
$50,000 or more	58%	47%	
Relationship Status (%)			≤.001^4^
Never married	16%	54%	
Living with partner	9%	19%	
Married	45%	19%	
Divorced or separated	22%	7%	
Widowed	8%	<1%	
Country of Residence (%)			≤.001^5^
United States	69%	61%	
United Kingdom	9%	7%	
Canada	8%	6%	
Australia	5%	4%	
Other (%)	10%	21%	

^1^ Demographic characteristics (except age) were dichotomized and compared between Non-Drug Group and Psychedelic Group using Chi-square; age was compared using independent samples *t* tests.

^2^ *p* ≤0.001 indicates a significant difference between groups.

^3^ Proportion White relative to other categories combined.

^4^ Proportion never married relative to all other categories.

^5^ Proportion residing in the United States relative to all other countries combined.

Table 2 shows demographic information separately for each drug group. Relative to the other drug groups, the Ayahuasca group participants were significantly older (at the time of the survey and at the time of the experience) and had a significantly higher proportion of participants who were female, had a bachelor’s degree or higher in education, and were ever married or living with a partner. Other demographic characteristics were similar across drug groups. Relative to the Non-Drug group, the Ayahuasca group did not differ significantly in age at the time of the experience but was younger at the time of study participation.

**Table 2:** Participant characteristics for Non-Drug, Psilocybin, LSD, Ayahuasca, and DMT groups^1^^,^^2^.

	Non-Drug	Psilocybin	LSD	Ayahuasca	DMT
	*n* = 933	*n* = 766	*n* = 904	*n* = 282	*n =* 307
Mean age at study participation in years (SD)	55.2 (13.5)	**31.3 (12.2)** ^a^	**30.5 (14.8)** ^a^	**37.8 (11.6)** ^b^	**30.6 (12.9)** ^a^
Mean age at time of experience in years (SD)	32.0 (16.4)	**24.5 (9.1)** ^a^	**21.7 (6.7)** ^b^	34.6 (11.0)^c^	**26.6 (9.3)** ^a^
Sex (% male)	32%	**80%** ^a^	**80%** ^a^	**63%** ^b^	**82%** ^a^
Race (% White)	89%	86%^a^	84%^a^	83%^a^	83%^a^
Ethnicity (% Hispanic)	7%	9%^a^	11%^a^	13%^a^	9%^a^
Education (% Bachelor’s Degree or higher)	54%	**42%** ^a^	**38%** ^a^	62%^b^	**41%** ^a^
Annual household income (% less than $50,000)	42%	**54%** ^a^	**52%** ^a^	47%^b^	**57%** ^a^
Relationship Status (% never married)	16%	**51%** ^a^	**62%** ^b^	**36%** ^c^	**58%** ^a,b^
Country of Residence (% United States)	69%	62%^a^	68%^a^	**40%** ^b^	63%^a^

^1^ Dichotomous demographic variables were analyzed with a general linear model with a logit link. Age was analyzed with ANOVA. Pairwise comparisons among groups were adjusted using Bonferroni method to control for Type I error.

^2^ Bold font = significant difference from the Non-Drug Group (p≤0.001); drug groups not sharing a common letter are significantly different (p≤0.001).

Additional descriptive information about participant religious orientation and beliefs about the afterlife are presented in S1 and S2 Tables in S1 File. Religious orientation and afterlife beliefs are presented descriptively because the survey did not determine whether these beliefs were present before or were affected by the psychedelic or near-death experience.

### Circumstances of the experience

Table 3 shows the circumstances of the experience for the Non-Drug Group and the Psychedelic Group. Participants in the Psychedelic Group were more likely to report that their experience lasted an hour or more (66%) relative to the Non-Drug Group (31%). Participants in the Non-Drug Group were more likely to report a very brief experience lasting five minutes or less (40%) relative to the Psychedelic Group (7%). The DMT group was more likely to report a shorter duration of experience relative to the other drug groups (Table 4).

**Table 3:** Circumstances of the experience in the Non-Drug Group and the Psychedelic Group.

	Non-Drug	Psychedelic	Regression Analyses^1^^,^^2^	
	*n* = 933	*n* = 2259	*OR*	*p* value
Duration of Experience (%)				
Five minutes or less	40%	**7%**	0.15	≤.001
Between 5 minutes and 1 hour	29%	27%	0.87	ns
1 hour or more	31%	**66%**	4.00	≤.001
Medically unconscious at any time during the experience (i.e., completely unresponsive to verbal or physical stimuli) (%)	36%	**10%**	0.15	≤.001
Clinically dead (i.e., cessation of breath, heart function) (%)	21%	**<1%**	0.01	≤.001
Medical professional confirmed to be clinically dead (%)	11%	**<1%**	0.004	≤.001
Imminent Danger (%)				
Life was in danger and believed it to be at the time	28%	**2%**	0.40	≤.001
Life was in danger but did not believe it to be at the time	19%	**1%**	0.41	≤.001
Life was not in danger but believed it to be at the time	6%	**20%**	2.11	≤.001
Life was not in danger and did not believe it to be at the time	48%	**77%**	5.77	≤.001

^1^ Each row presents an individual logistic regression analysis for each outcome with group as a factor while controlling for demographic variables as described in text. Coefficients for group are presented as odds ratio (*OR*).

^2^ Bold font data in the Psychedelic Group indicates a significant difference from Non-Drug Group (p≤0.001).

**Table 4:** Circumstances of the experience in the Non-Drug, Psilocybin, LSD, Ayahuasca, and DMT groups^1^^,^^2^.

	Non-Drug	Psilocybin	LSD	Ayahuasca	DMT
	*n* = 933	*n* = 766	*n* = 904	*n* = 282	*n* = 307
Duration of Experience (%)					
Five minutes or less	40%	**5%** ^a,b^	**8%** ^a,c^	**2%** ^b^	**16%** ^c^
Between 5 minutes and 1 hour	29%	23%^a^	20%^a^	21%^a^	**61%** ^b^
1 hour or more	31%	**71%** ^a^	**72%** ^a^	**77%** ^a^	23%^b^
Medically unconscious at any time during the experience (i.e., completely unresponsive to verbal or physical stimuli) (%)	36%	**5%** ^a^	**9%** ^a^	**7%** ^a^	31%^b^
Clinically dead (i.e., cessation of breath, heart function) (%)	21%	**<1%** ^a^	**<1%** ^a^	**<1%** ^a^	**<1%** ^a^
Medical professional confirmed to be clinically dead (%)	11%	**0%** ^a^	**0%** ^a^	**0%** ^a^	**0%** ^a^
Imminent Danger (%)					
Life was in danger and believed it to be at the time	28%	**1%** ^a,b^	**3%** ^a^	**2%** ^a,b^	**0%** ^b^
Life was in danger but did not believe it to be at the time	19%	**1%** ^**a**^	**1%** ^a^	**0%** ^a^	**0%** ^a^
Life was not in danger but believed it to be at the time	6%	20%^a^	21%^a^	**22%** ^a^	19%^a^
Life was not in danger and did not believe it to be at the time	48%	**78%** ^a^	**75%** ^a^	**76%** ^a^	**81%** ^a^

^1^ Comparisons between drug groups were made using general linear model with a logit link and Type III sums of squares including covariates as described in text; pairwise comparisons were adjusted using the Bonferroni method to control for Type I error rate.

^2^ Bold font = significant difference from the Non-Drug Group (p≤0.001); drug groups not sharing a common letter are significantly different (p≤0.001).

It was more common for participants in the Non-Drug Group to report being medically unconscious during the experience (i.e., completely unresponsive to verbal or physical stimuli) (36%) relative to the Psychedelic Group as a whole (10%) (Table 3). Medical unconsciousness was significantly more prevalent among the DMT group (31%) relative to the other drug groups (<10%) and comparable to the Non-Drug Group (Table 4). Twenty-one percent of the Non-Drug Group reported that they were clinically dead (i.e., cessation of breath, heart function) during the experience, and a further 11% reported that a medical professional confirmed them to be clinically dead at the time of the experience, whereas less than 1% of any drug group reported clinical death at the time of the experience (Table 4).

The Non-Drug Group was more likely to report that their life was in danger at the time of the experience (47%) relative to the Psychedelic Group (3%), and most Psychedelic respondents neither experienced danger to their life nor believed their life was in danger at the time of the experience (77%) (Table 3). A similar pattern was observed across all the drug groups (Table 4).

Further information about the Non-Drug Group not shown in the tables is that 46% of respondents from this group categorized their experience as a “near-death” experience, whereas 54% categorized their experience as an “other non-ordinary” experience. Examples of the kinds of experiences reported by participants in the Non-Drug Group in the narrative response included near-death experiences, out-of-body experiences, the respondent’s experience of the death of close friends or family, and communication with those who have died. About half of those in the Non-Drug Group identifying their experience as a “near-death” experience (50%) or “other non-ordinary” experience (43%) indicated that their life was in imminent danger at the time of the experience.

### Mystical-type and other subjective features of the experience

Table 5 shows the Non-Drug Group and Psychedelic Group data for the Mystical Experience Questionnaire-30 (MEQ-30), Greyson Near-Death Experience Questionnaire (Greyson NDE Scale), and other subjective features of the experience. Table 6 shows these measures across all the drug groups.

**Table 5:** Mystical-type and other subjective features of the experience among the Psychedelic Group and the Non-Drug Group.

	Non-Drug	Psychedelic	Regression Analyses^1^^,^^2^	
	*n* = 933	*n* = 2259	*β* / *OR*	*p* value
Mystical Experience Questionnaire (MEQ30)				
Mean proportion of max scores for each factor and total (SD)			*β*	
Mystical	.67 (.29)	**.76 (.20)**	.71	≤.001
Positive mood	.70 (.29)	**.75 (.22)**	.45	≤.001
Transcendence of time and space	.62 (.33)	**.70 (.25)**	.39	≤.001
Ineffability	.78 (.27)	**.85 (.18)**	.27	≤.001
Total Score	.68 (.27)	**.76 (.17)**	.65	≤.001
Percent fulfilling criteria for complete mystical experience			*OR*	
Complete mystical experience	47%	**55%**	2.08	≤.001
Near-Death Experience Questionnaire (Greyson NDE Scale)				
Mean proportion of max scores for each subscale and total (SD)			*β*	p
Cognitive	.38 (.28)	**.57 (.22)**	.60	≤.001
Affective	.58 (.34)	**.67 (.28)**	.50	≤.001
Paranormal	.38 (.25)	**.45 (.23)**	.30	≤.001
Transcendental	.42 (.32)	.34 (.30)	-.12	ns
Total Score	.44 (.23)	**.51 (.18)**	.42	≤.001
States of Consciousness Questionnaire Items (SOCQ)				
Mean score for each item (range: 0 = none/not at all; 5 = extreme) (SD)			*β*	
Feeling of being reborn	1.83 (2.06)	**2.80 (1.89)**	.36	≤.001
Convincing feeling of reliving biological birth	0.47 (1.26)	**0.86 (1.50)**	.25	≤.001
Reliving of situations and events from your childhood	0.96 (1.73)	**1.50 (1.75)**	.20	≤.001
Déjà vu (experienced exact situation, but no real memory of it)	0.90 (1.42)	**1.43 (1.50)**	.19	≤.001
Convincing feelings of reliving a previous incarnation	0.86 (1.70)	1.14 (1.70)	.14	ns
Profound experience of your own death	2.05 (2.12)	2.54 (2.02)	.09	ns
Convincing feeling of obtaining information in an extrasensory manner	2.72 (2.21)	**1.80 (1.96)**	-.27	≤.001
Convincing feeling of contact with people who have died	2.26 (2.25)	**1.06 (1.68)**	-.44	≤.001
Percent endorsing an encounter with “God”			*OR*	
Encountered something that someone might call “God”	48%	**56%**	1.73	**≤.001**

^1^ Each row presents an individual regression analysis for each outcome with group as a factor while controlling for demographic variables as described in text. Coefficients for group are presented as standardized *β* for linear regressions and odds ratio (*OR*) for logistic regressions.

^2^ Bold font = group significant factor in regression analysis at *p* ≤.001.

**Table 6:** Mystical-type and other subjective features of the experience among the Non-Drug, Psilocybin, LSD, Ayahuasca, and DMT groups^1^^,^^2^.

	Non-Drug	Psilocybin	LSD	Ayahuasca	DMT
	*n* = 933	*n* = 766	n = 904	n = 282	n = 307
Mystical Experience Questionnaire (MEQ30)					
Mean proportion of max scores for each factor and total (SD)					
Mystical	.67 (.29)	**.76 (.20)** ^a,b^	**.74 (.21)** ^a^	**.82 (.18)** ^b^	**.80 (.19)** ^b^
Positive mood	.70 (.29)	**.74 (.22)** ^a^	**.73 (.23)** ^a^	**.80 (.20)** ^a^	**.78 (.22)** ^a^
Transcendence of time and space	.62 (.33)	**.66 (.24)** ^a^	**.66 (.26)** ^a^	**.77 (.22)** ^b^	**.84 (.18)** ^c^
Ineffability	.78 (.27)	.83 (.19)^a^	.84 (.18)^a^	**.87 (.16)** ^a,b^	**.90 (.15)** ^b^
Total Score	.68 (.26)	**.74 (.17)** ^a^	**.73 (.18)** ^a^	**.81 (.15)** ^b^	**.82 (.15)** ^b^
Percent fulfilling criteria for complete mystical experience					
Complete mystical experience	47%	**51%** ^**a**^	48%^a^	**68%** ^**b**^	**73%** ^**b**^
Near-Death Experience Questionnaire (Greyson NDE Scale)					
Mean proportion of max scores for each factor and total (SD)					
Cognitive	.38 (.28)	**.56 (.22)** ^a^	**.59 (.22)** ^a^	**.57 (.23)** ^a^	**.55 (.22)** ^a^
Affective	.58 (.34)	**.67 (.28)** ^a,b^	**.64 (.27)** ^a^	**.73 (.26)** ^a,b^	**.73 (.29)** ^b^
Paranormal	.38 (.25)	**.44 (.23)** ^a,b^	.44 (.22)^a^	**.49 (.23)** ^b^	**.49 (.21)** ^a,b^
Transcendental	.42 (.32)	**.29 (.28)** ^a^	**.25 (.27)** ^a^	**.51 (.27)** ^b^	**.56 (.27)** ^b^
Total Score	.44 (.23)	**.49 (.18)** ^a^	**.48 (.17)** ^a^	**.57 (.17)** ^b^	**.58 (.17)** ^b^
States of Consciousness Questionnaire Items (SOCQ)					
Mean score for each item (range 0 none/not at all—5 extreme) (SD)					
Feeling of being reborn	1.83 (2.06)	**2.71 (1.85)** ^a,b^	**2.65 (1.93)** ^a^	**3.16 (1.81)** ^c^	**3.15 (1.83)** ^b,c^
Convincing feeling of reliving biological birth	0.47 (1.26)	0.84 (1.48)^a^	0.76 (1.43)^a^	**1.09 (1.66)** ^a^	**0.99 (1.59)** ^a^
Reliving of situations and events from your childhood	0.96 (1.73)	1.56 (1.73)^a^	1.51 (1.73)^a^	**1.96 (1.91)** ^b^	0.87 (1.51)^c^
Déjà vu (experienced exact situation, but no real memory of it)	0.90 (1.42)	1.37 (1.44)^a^	1.43 (1.47)^a^	1.32 (1.53)^a^	**1.67 (1.68)** ^a^
Convincing feelings of reliving a previous incarnation	0.86 (1.70)	1.06 (1.61)^a^	1.51 (1.74)^a^	1.35 (1.83)^a^	1.09 (1.68)^a^
Profound experience of your own death	2.05 (2.12)	2.41 (2.0)^a^	2.44 (2.05)^a^	2.79 (1.98)^a^	2.93 (1.99)^a^
Convincing feeling of obtaining information in an extrasensory manner	2.72 (2.21)	**1.77 (1.93)** ^a,b^	**1.65 (1.92)** ^a^	2.34 (2.00)^b^	**1.79 (2.03)** ^a^
Convincing feeling of contact with people who have died	2.26 (2.25)	**0.96 (1.58)** ^a^	**0.88 (1.57)** ^a^	1.73 (1.96)^b^	**1.20 (1.76)** ^a,b^
Percent endorsing an encounter with “God”					
Encountered something that someone might call “God”	48%	54%^a^	49%^a^	**71%** ^b^	**68%** ^b^

^1^ Comparisons between drug groups for dichotomous outcomes were made using general linear model with a logit link and Type III sums of squares including covariates as described in text; continuous data were analyzed using ANOVA with the same covariates and Type III Sums of Squares; pairwise comparisons were adjusted using the Bonferroni method to control for Type I error rate.

^2^ Bold font = significant difference from the Non-Drug Group (p≤0.001); drug groups not sharing a common letter are significantly different (p≤0.001).

#### MEQ-30

Total and subscale scores on the MEQ exceeded 50% of maximum possible scores in both the Non-Drug and Psychedelic groups and it was common for participants in both groups to fulfill the *a priori* criteria for a “complete” mystical experience according to the MEQ-30, with 55% in the Psychedelic Group and 47% in the Non-Drug Group meeting this threshold (Table 5). However, across all these measures, the Psychedelic Group was significantly higher than the Non-Drug Group. When examined separately across drug groups (Table 6), the percentage of those in the LSD (48%) and Psilocybin (51%) groups meeting criteria for a “complete” mystical experience were comparable to the Non-Drug Group (47%), whereas it was significantly more common to meet criteria for a “complete” mystical experience in the Ayahuasca (68%) and DMT (73%) groups. On the subscale assessing transcendence of time and space, the Ayahuasca and DMT groups had higher mean proportion of subscale score relative to the LSD and Psilocybin groups, whose mean proportion subscale scores were closer to the Non-Drug Group (Table 6).

#### Greyson NDE scale

On the *cognitive* subscale of the Greyson NDE Scale, which contained items assessing whether participants experienced speeded up time or thoughts, saw scenes from the past, or experienced sudden understanding, the mean proportion of subscale score was higher in the Psychedelic Group relative to the Non-Drug Group. The Psychedelic Group also had higher scores than the Non-Drug Group on the *affective* and *paranormal* subscales and the overall total on the NDE Scale, but the magnitude of these differences were smaller relative to the difference on *cognitive* subscale (Table 5). This pattern was generally consistent across all the drug groups (See Table 6). On the *transcendental* subscale of the Greyson NDE Scale, which contained items assessing whether participants seemed to enter an “other, unearthly world,” encountered a “mystical being or presence”, saw “deceased spirits or religious figures,” and came to “a border or point of no return”, the Non-Drug Group was intermediate to the drug groups (Table 6). Specifically, the Psilocybin and LSD Groups had significantly lower proportion of total scores on the *transcendental* NDE subscale relative to the Non-Drug Group, and the DMT and Ayahuasca groups had significantly higher proportion total scores.

Proportion total scores on the NDE Scale and the MEQ were positively and significantly correlated for the total sample (*r =* .77, *n =* 3192, *p* ≤0.001). The positive association between the NDE Scale and the MEQ was present in both the Non-Drug Group (*r* = .82, *n =* 933, *p* ≤0.001) and the Psychedelic Group (*r =* .72, *n* = 2259, *p* ≤0.001).

### Other subjective features

Both the Non-Drug and the Psychedelic Groups reported similar mean ratings for feeling a “profound experience of your own death”, where the mean ratings were in the slight to moderate range (e.g., mean ratings 2–3 on a 5-point scale) (Table 5). Participants in all the Psychedelic Groups reported significantly higher ratings for the extent to which they experienced feelings of being reborn (means range = 2.7–3.2 on a 5-point scale) relative to the Non-Drug Group (mean = 1.8) (Table 6). The Non-Drug Group had significantly higher ratings for convincing feelings of contact with people who have died, and convincing feelings of obtaining information in an extrasensory manner relative to the Psychedelic Group (Table 5). The Ayahuasca Group had higher ratings for feelings of obtaining information in an extrasensory manner relative to the other drug groups, where the Ayahuasca Group ratings were not significantly different than ratings for the Non-Drug Group (Table 6). For feelings of contact with people who have died, the Ayahuasca and DMT groups had higher ratings relative to the Psilocybin and LSD groups. For reliving situations from childhood, the Ayahuasca Group had higher ratings relative to the other drug groups, where the DMT Group had the lowest ratings among the groups and the Psilocybin, LSD, and Non-Drug groups’ mean ratings were intermediate to the Ayahuasca and DMT groups. Participants in the Ayahuasca and DMT Groups were significantly more likely to report encountering “something that someone might call “God” e.g., the God of your understanding” during the experience relative to the Psilocybin, LSD, and Non-Drug groups.

### Changes in fear of death and death attitudes from before to after the experience

A large majority of participants in both groups (88% of the Non-Drug Group and 89% of the Psychedelic Group) reported that their experience resulted in a decrease in their fear of death (Table 7). Only 5% of the Non-Drug Group and 6% of the Psychedelic Group reported that the experience resulted in an increase in their fear of death. Similar large majorities of the Psychedelic Group and Non-Drug Group reported that the experience resulted in positive, desirable changes in their curiosity and interest in death, attitudes about the death of others, and attitudes about their own death. This was also true when each drug group was considered separately (Table 8).

**Table 7:** Changes in death attitudes attributed to the experience among the Psychedelic Group and the Non-Drug Group.

	Non-Drug	Psychedelic	Regression Analyses^1^^,^^2^	
	*n* = 933	*n* = 2259	*β* / *OR*	*p* value
Changes in fear of death (%)			*OR*	
Decreased fear of death	88%	89%	1.29	ns
Increased fear of death	5%	6%	0.53	ns
Curiosity or interest in death (%)			*OR*	
Positive, desirable change	84%	82%	0.82	ns
Negative, detrimental change	2%	2%	0.49	ns
Attitudes about death of others (%)			*OR*	
Positive, desirable change	87%	85%	1.26	ns
Negative, detrimental change	3%	2%	0.43	ns
Attitudes about own death (%)			*OR*	
Positive, desirable change	90%	92%	1.35	ns
Negative, detrimental change	3%	3%	0.40	ns
Death Attitudes Profile				
Mean change score^3^ for each factor (SD)			*β*	
Fear of death (e.g., “I have an intense fear of death.”)	-2.32 (1.80)	-2.13 (1.66)	.08	ns
Neutral acceptance (e.g., “Death is neither good nor bad.”)	0.95 (1.22)	0.90 (1.03)	-.11	ns
Death avoidance (e.g., “I avoid death thoughts at all costs.”)	-2.01 (1.86)	**-1.43 (1.68)**	.26	≤.001
Approach acceptance (e.g., “I look forward to life after death.”)	1.13 (1.46)	**0.57 (1.27)**	-.36	≤.001
Escape acceptance (e.g., “I see death as a relief from the burden of this life.”)	0.27 (1.65)	**-0.42 (1.57)**	-.34	≤.001

^1^ Each row presents an individual regression analysis for each outcome with group as a factor while controlling for demographic variables as described in text. Coefficients for group are presented as standardized *β* for linear regressions and odds ratio (*OR*) for logistic regressions.

^2^ Bold font = group significant factor in regression analysis at *p* ≤.001.

^3^ Participants rated attitudes before and after the experience on a 7-point Likert scale; negative change scores indicate a decrease in the rating on that scale from before to after and positive change scores indicate an increase from before to after.

**Table 8:** Changes in death attitudes attributed to the experience among the Non-Drug, Psilocybin, LSD, Ayahuasca, and DMT groups^1^^,^^2^.

	Non-Drug	Psilocybin	LSD	Ayahuasca	DMT
	*n* = 933	*n* = 766	*n* = 904	*n* = 282	*n* = 307
Changes in fear of death (%)					
Decreased fear of death	88%	91%^a^	86%^a^	90%^a^	90%^a^
Increased fear of death	5%	6%^a^	8%^a^	5%^a^	5%^a^
Curiosity or interest in death (%)					
Positive, desirable change	84%	84%^a^	80%^a^	84%^a^	81%^a^
Negative, detrimental change	2%	2%^a^	3%^a^	2%^a^	2%^a^
Attitudes about death of others (%)					
Positive, desirable change	87%	86%^a^	84%^a^	90%^a^	82%^a^
Negative, detrimental change	3%	2%^a^	3%^a^	1%^a^	4%^a^
Attitudes about own death (%)					
Positive, desirable change	90%	92%^a^	92%^a^	94%^a^	93%^a^
Negative, detrimental change	3%	2%^a^	3%^a^	2%^a^	3%^a^
Death Attitudes Profile					
Mean change score^3^ for each factor (SD)					
Fear of death (e.g., “I have an intense fear of death.”)	-2.32 (1.80)	-2.13 (1.65)^a^	-2.14 (1.70)^a^	-1.97 (1.65)^a^	-2.21 (1.62)^a^
Neutral acceptance (e.g., “Death is neither good nor bad.”)	0.95 (1.22)	0.91 (1.05)^a^	0.95 (1.07) ^a^	0.76 (.87)^a^	0.87 (1.00)^a^
Death avoidance (e.g., “I avoid death thoughts at all costs.”)	-2.01 (1.86)	**-1.45 (1.71)** ^a^	**-1.46 (1.75)** ^a^	**-1.30 (1.61)** ^a^	**-1.39 (1.49)** ^a^
Approach acceptance (e.g., “I look forward to life after death.”)	1.13 (1.46)	**0.58 (1.21)** ^a,b^	**0.42 (1.32)** ^a^	**0.68 (1.15)** ^a,b^	0.91 (1.31)^b^
Escape acceptance (e.g., “I see death as a relief from the burden of this life.”)	0.27 (1.65)	**-0.33 (1.49)** ^a^	**-0.45 (1.63)** ^a^	**-0.66 (1.70)** ^a^	-0.30 (1.43)^a^

^1^ Comparisons between drug groups for dichotomous outcomes were made using general linear model with a logit link and Type III sums of squares including covariates as described in text; continuous data were analyzed using ANOVA with the same covariates and Type III Sums of Squares; pairwise comparisons were adjusted using the Bonferroni method to control for Type I error rate.

^2^ Bold font = significant difference from the Non-Drug Group (p≤0.001); drug groups not sharing a common letter are significantly different (p≤0.001).

^3^ Participants rated attitudes before and after the experience on a 7-point Likert scale; negative change scores indicate a decrease in the rating on that scale from before to after and positive change scores indicate an increase from before to after.

Tables 7 and 8 show before to after the experience difference scores on each of five factors for each group on the Death Attitudes Profile. In the description of results below, change scores are expressed as percentage of maximum possible change scores on the Likert rating scale to facilitate understanding of the magnitudes of effects. When rating attitudes before and after the experience, participants in both the Non-Drug and Psychedelic groups reported similar decreases in agreement (36–39%) on the *fear of death* subscale (e.g., “I have an intense fear of death.”) of the Death Attitudes Profile (Tables 7 and 8). There was little reported change across any group (13–16% increase) for the *neutral acceptance* subscale (e.g., “Death is neither good nor bad”) of the Death Attitudes Profile. The Non-Drug Group reported a significantly greater decrease (34%) in *death avoidance* (e.g., “I avoid death thoughts at all costs”) relative to the drug groups (22–24% decrease). The Non-Drug Group reported modest increases (19%) in *approach acceptance* (e.g., “I look forward to life after death”) resulting from their experience relative to the drug groups (7–15% of Likert scale). Neither the Non-Drug or any drug group showed consistent increases or decreases (i.e., only mean change >10% was 11% decrease in Ayahuasca Group) for *escape acceptance* (e.g., “I see death as a relief from the burden of this life”) resulting from their experience.

### Comparison of experience relative to other lifetime experiences

Participants in all the groups reported high ratings with respect to how personally meaningful, spiritually significant, and psychologically insightful the experience was relative to other lifetime experiences (Tables 9 and 10). Significantly more participants in the Non-Drug Group rated the experience as the single most personally meaningful, spiritually significant, personally psychologically insightful, and psychologically challenging the experience of their lifetime relative to the Psychedelic Group. Notably, in contrast to the other drug groups, the Ayahuasca Group was never significantly different from the Non-Drug Group for ratings of the experience relative to other lifetime experiences on these dimensions.

**Table 9:** Comparison of experience relative to other lifetime experiences among the Psychedelic Group and the Non-Drug Group.

	Non-Drug	Psychedelic	Regression Analyses^1^^,^^2^	
	*n* = 933	*n* = 2259	*β* / *OR*	*p* value
Mean rating relative to other lifetime experiences (range 1–8)^3^			*β*	
How personally meaningful was the experience	7.2 (1.1)	**6.9 (1.0)**	-.23	≤.001
How spiritually significant was the experience	7.1 (1.4)	6.8 (1.5)	-.16	ns
How personally psychologically insightful was the experience	6.8 (1.8)	6.7 (1.4)	-.01	ns
How psychologically challenging was the experience	5.4 (2.7)	5.5 (2.2)	-.10	ns
Percent rating the experience as top 5 most of lifetime			*OR*	
How personally meaningful was the experience	85%	75%	0.66	ns
How spiritually significant was the experience	84%	**76%**	0.65	≤.001
How personally psychologically insightful was the experience	78%	72%	0.82	ns
How psychologically challenging was the experience	54%	**47%**	0.66	≤.001
Percent rating the experience as single most of lifetime			*OR*	
How personally meaningful was the experience	46%	**25%**	0.42	≤.001
How spiritually significant was the experience	50%	**38%**	0.51	≤.001
How personally psychologically insightful was the experience	41%	**29%**	0.54	≤.001
How psychologically challenging was the experience	28%	**18%**	0.44	≤.001

^1^ Each row presents an individual regression analysis for each outcome with group as a factor while controlling for demographic variables as described in text. Coefficients for group are presented as standardized *β* for linear regressions and odds ratio (*OR*) for logistic regressions.

^2^ Bold font = group significant factor in regression analysis at *p* ≤.001.

^3^ Rating options ranged from 1 = no more than routine, everyday experience; 5 = similar to experiences that occur on average once every 5 years; 6 = among the 10 most in my life; 7 = among the 5 most of my life; 8 = the single most of my life.

**Table 10:** Comparison of experience relative to other lifetime experiences among the Non-Drug, Psilocybin, LSD, Ayahuasca, and DMT groups^1^^,^^2^.

	Non-Drug	Psilocybin	LSD	Ayahuasca	DMT
	*n* = 933	*n* = 766	*n* = 904	*n* = 282	*n* = 307
Mean rating relative to other lifetime experiences (range 1–8)^3^					
How personally meaningful was the experience	7.2 (1.1)	**6.8 (1.0)** ^a^	**6.8 (1.1)** ^a^	7.1 (1.0)^b^	7.0 (.9)^a,b^
How spiritually significant was the experience	7.1 (1.4)	6.8 (1.5)^a^	**6.7 (1.7)** ^a^	7.2 (1.1)^b^	7.0 (1.5)^a,b^
How personally psychologically insightful was the experience	6.8 (1.8)	6.7 (1.3)^a^	6.7 (1.4)^a^	7.0 (1.2)^a^	6.6 (1.6)^a^
How psychologically challenging was the experience	5.4 (2.7)	5.3 (2.2)^a^	5.6 (2.2)^a,b^	5.9 (2.2)^b^	5.6 (2.2)^a,b^
Percent rating the experience as top 5 most of lifetime					
How personally meaningful was the experience	85%	**71%** ^a^	**73%** ^a^	87%^b^	78%^a,b^
How spiritually significant was the experience	84%	**75%** ^a^	**73%** ^a^	87%^b^	80%^a,b^
How personally psychologically insightful was the experience	78%	70%^a^	71%^a^	79%^a^	70%^a^
How psychologically challenging was the experience	54%	**41%** ^**a**^	49%^a,b^	58%^b^	**44%** ^a,b^
Percent rating the experience as single most of lifetime					
How personally meaningful was the experience	46%	**21%** ^a^	**24%** ^a,b^	34%^b^	**28%** ^a,b^
How spiritually significant was the experience	50%	**37%** ^a^	**35%** ^a^	45%^a^	46%^a^
How personally psychologically insightful was the experience	41%	**28%** ^a^	**30%** ^a^	34%^a^	**29%** ^a^
How psychologically challenging was the experience	28%	**13%** ^a^	**19%** ^a^	21%^a^	**20%** ^a^

^1^ Comparisons between drug groups for dichotomous outcomes were made using general linear model with a logit link and Type III sums of squares including covariates as described in text; continuous data were analyzed using ANOVA with the same covariates and Type III Sums of Squares; pairwise comparisons were adjusted using the Bonferroni method to control for Type I error rate.

^2^ Bold font = significant difference from the Non-Drug Group (p≤0.001); drug groups not sharing a common letter are significantly different (p≤0.001).

^3^ Rating options ranged from 1 = no more than routine, everyday experience; 5 = similar to experiences that occur on average once every 5 years; 6 = among the 10 most in my life; 7 = among the 5 most of my life; 8 = the single most of my life.

### Persisting changes attributed to the experience

Participants in all the groups reported similar mean ratings for long-term and persisting changes because of the experience, most often indicating moderate positive and desirable changes in personal wellbeing or life satisfaction, sense of their life’s purpose, sense of their life’s meaning, social relationships, mood, and spirituality (Tables 11 and 12).

**Table 11:** Persisting changes attributed to the experience among the Non-Drug and Psychedelic groups.

	Non-Drug	Psychedelic	Regression Analyses^1^^,^^2^	
	n = 933	n = 2259	*β*	*p* value
Overall life changes (range from –3 to +3)^3^				
Personal well-being or life satisfaction	2.3 (1.1)	**2.3 (1.1)**	**.18**	≤.001^4^
Life’s purpose	2.1 (1.2)	2.0 (1.2)	.03	ns
Life’s meaning	2.1 (1.2)	2.1 (1.1)	.04	ns
Social relationships as a whole	1.7 (1.4)	1.7 (1.3)	.16	ns
Mood	1.7 (1.3)	**1.7 (1.2)**	**.19**	≤.001^4^
Behavioral changes	1.7 (1.2)	1.7 (1.2)	.10	ns
How spiritual you are	2.1 (1.2)	2.0 (1.2)	-.15	ns

^1^ Each row presents an individual linear regression analysis for each outcome with group as a predictor while controlling for demographic variables as described in text. Coefficients for group are presented as standardized β.

^2^ Bold font = group significant factor in regression analysis at *p* ≤.001

^3^ Rating options ranged from -3 = Strong negative change that I consider undesirable to +3 = Strong positive change that I consider desirable, with 0 = no change.

^4^ The significant group effect in regression models despite similar means can be explained by the covariate adjustment for age. Age was systematically lower in the Psychedelic Group, but also associated with higher ratings of positive changes in mood and in well-being or life satisfaction. Thus, while controlling for age, participants in the Psychedelic group had significantly higher ratings of positive changes in in well-being or life satisfaction and mood.

**Table 12:** Persisting changes attributed to the experience among the Non-Drug, Psilocybin, LSD, Ayahuasca, and DMT groups^1^^,^^2^.

	Non-Drug	Psilocybin	LSD	Ayahuasca	DMT
	*n* = 933	*n* = 766	*n* = 904	*n* = 282	*n* = 307
Overall life changes (range from –3 to +3)^3^					
Personal well-being or life satisfaction	2.4 (1.3)	2.3 (1.1)^a^	2.2 (1.2)^a^	**2.5 (0.9)** ^a^	2.4 (0.9)^a^
Life’s purpose	2.2 (1.2)	2.0 (1.1)^a^	2.0 (1.2)^a^	2.2 (1.1)^a^	2.0 (1.2)^a^
Life’s meaning	2.3 (1.2)	2.1 (1.1)^a^	2.0 (1.2)^a^	2.3 (1.0)^a^	2.1 (1.2)^a^
Social relationships as a whole	1.8 (1.6)	1.7 (1.2)^a,b^	1.6 (1.4)^a^	**2.1 (1.1)** ^b^	1.8 (1.2)^a,b^
Mood	1.7 (1.5)	1.7 (1.2)^a,b^	1.6 (1.3)^a^	**2.0 (1.0)** ^b^	1.8 (1.2)^a,b^
Behavioral changes	1.8 (1.4)	1.7 (1.2)^a^	1.6 (1.2)^a^	**2.0 (1.0)** ^b^	1.7 (1.2)^a,b^
How spiritual you are	2.4 (1.1)	2.0 (1.1)^a^	**1.9 (1.2)** ^a^	2.2 (1.0)^a^	2.0 (1.2)^a^

^1^ Comparisons between drug groups were analyzed using ANOVA covariates as described in text and Type III Sums of Squares; pairwise comparisons were adjusted using the Bonferroni method to control for Type I error rate.

^2^ Bold font = significant difference from the Non-Drug Group (p≤0.001); drug groups not sharing a common letter are significantly different (p≤0.001)

^3^ Rating options ranged from -3 = Strong negative change that I consider undesirable to +3 = Strong positive change that I consider desirable, with 0 = no change.

## Discussion

The present study provides the most comprehensive and detailed comparison to date of psychedelic-occasioned and non-drug experiences associated with enduring changes in attitudes and beliefs about death and dying. Across multiple measures, approximately 90% of survey respondents in both the drug and non-drug groups reported that the experience decreased their fear of death and resulted in positive, desirable changes in their curiosity and attitudes about death, including their own death. Fewer than 1 in 10 survey respondents reported any increased fear of death or negative changes in death attitudes. Participants with both drug-occasioned and non-drug experiences reported similar high ratings for the personal meaning, spiritual significance, and psychological insightfulness of the experience, with participants in the non-drug group being more likely to rate the experience as the single most meaningful, spiritually significant, and insightful experience of their lifetime.

In the psychedelic group, these decreases in fear of death and positive attributions of the experience are consistent with other cross-sectional survey data. Previous survey studies of psychedelic-occasioned “God encounter” experiences and DMT-occasioned encounters with a “seemingly autonomous entity” had similar high ratings for personal meaning, spiritual significance, and psychological insight as the present study, and respondents noted positive desirable changes in attitudes about death [21, 22]. In contrast to these prior surveys, the present study enrolled individuals who endorsed having had either a non-drug or psychedelic occasioned experience that changed their beliefs about death and the study provides much greater detail about the nature of changes in attitudes about death.

The present data are also consistent with existing literature examining the effects of psychedelics among patients with life-threatening disease in randomized double-blind trials [16–20]. Decreased death anxiety has been suggested as a potentially important mechanism for the positive enduring clinical effects of psychedelic drugs across diagnoses and experiences, where a death-like experiences may contribute to a decrease in fear of death and lead to positive therapeutic changes [45].

The present data are also consistent with reports suggesting that non-drug-associated near-death and other non-ordinary experiences result in decreased fear of death and other positive changes in death attitudes [29, 34, 35]. Some recent prospective experimental data also support the potential for near-death and out-of-body experiences to change perspective on death and dying. Specifically, study participants who experienced experimenter-generated immersive virtual reality environments reported positive life changes following a virtual near-death experience and decreased fear of death following a virtual out-of-body experience [46, 47]. Future investigation of other novel paradigms for occasioning near-death and related experiences with and without psychedelics seems likely to be of value.

Because of the large number of outcome measures and complexity of the results, the remainder of this Discussion section will first summarize the most salient similarities and differences between the non-drug and the psychedelic-occasioned experiences followed by a summary of comparisons among the four psychedelic groups.

### Similarities and differences between non-drug and psychedelic-occasioned experiences

Both the non-drug and psychedelic participants showed robust increases on widely used measures assessing mystical and near-death experiences which have previously defined thresholds for effect. Specifically, on the Mystical Experience Questionnaire, mean total scores expressed as a percent of total possible score of .68 (Non-Drug Group) and .76 (Psychedelic Group) are in the range of scores produced by high doses of psilocybin (20 to 30 mg/70kg) administered in the laboratory and much higher than those after placebo [7]. Likewise, the percentage of participants meeting criteria for having a complete mystical experience in the Non-Drug Group (47%) and Psychedelic Group (55%) were in the range produced by 10 mg/70 kg (low dose) to 20 mg/70 kg psilocybin (moderate dose) administered in the laboratory [7]. With regard to the Greyson Near-Death Experience Scale, the mean total scores expressed as a percent of total possible score of .44 (Non-Drug Group) and .51 (Psychedelic Group) are well above the threshold value of .22 (a score of 7 of 32) that is considered a threshold value for defining a near-death experience [28]. Although both groups had high total scores on both the mystical and near-death experience questionnaires, almost all ratings in the Psychedelic Group were significantly higher than those in the Non-Drug Group.

With regard to some specific phenomenological features of the experience, about half of both groups endorsed having "Encountered something someone might call ‘*God*‴, although this was significantly greater in Psychedelic Group than the Non-Drug Group (56% vs. 48%). Among ratings with effect sizes >.25, the rating of "Feeling of being reborn" was significantly higher in the Psychedelic Group, whereas "Convincing feelings of obtaining information in an extrasensory manner” and “feelings of contact with people who have died” were significantly higher in the Non-Drug Group.

With regard to changes attributed to the experience, both groups showed similar high rates of endorsing decreased fear of death and other positive, desirable changes in death attitudes. Likewise, both groups provided high ratings of the personal meaning, spiritual significance, and psychological insight attributed to the experience however the Non-Drug Group were more likely to rate their experience as the single such experience of their lifetime.

**Fig 1:**
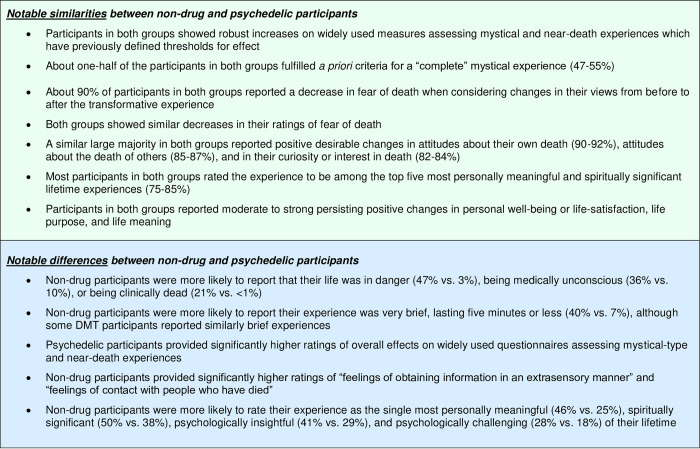
Similarities and differences in experiences between non-drug and psychedelic participants. Summary of notable similarities and differences in the circumstances of the experience, the subjective features of the experience, and the persisting changes attributed to the experience between the Non-Drug Group (naturally occurring experiences) and the combined Psychedelic Group (psychedelic-occasioned experiences).

Fig 1 presents a summary of the most notable similarities and differences between the Non-Drug Group and the Psychedelic Group.

### Similarities and differences among different psychedelics

#### Psilocybin and LSD groups were very similar

There were some small but significant demographic differences between the Psilocybin and LSD groups with respect to age and relationship status, but the Psilocybin and LSD groups were not significantly different on any of the 62 outcome measures assessing the details and consequences of the experience. This finding is very similar to that reported in a previous survey study of "God encounter" experiences [21] and is interesting because, although psilocybin and LSD are both classic psychedelics whose primary effects are mediated at the 5HT2A receptor, they have different molecular structures, profiles of receptor activity, durations of action, with likely differences in functional potency and selectivity [48].

#### Ayahuasca group

Demographically, the Ayahuasca Group was the most unique of the psychedelic groups, being more likely to be older, female, college educated, married, not a U.S. resident, and have a higher income. The Ayahuasca Group had significantly higher scores than the Psilocybin and LSD groups on the mystical experience and near-death questionnaires and on several phenomenological features of the experience (e.g. feeling of reliving biological birth; an encounter with something someone might call "God"). Likewise, the Ayahuasca Group compared to the Psilocybin and LSD groups rated the experience to be more personally meaningful and spiritually significant and indicated significantly more positive life changes in social relationships, mood, and behavior.

#### DMT group

Demographically, the DMT Group differed from the Ayahuasca Group as described above but not from the Psilocybin and LSD groups. The DMT Group differed from all three other groups in reporting the shortest duration of experience, providing the lowest ratings that the experience involved reliving events from childhood, and were more likely to endorse complete unresponsiveness to verbal and physical stimuli during the experience relative to the other drug groups (though non-responsiveness was still reported by a minority of participants). Compared to the Psilocybin and LSD groups, the DMT Group was generally similar to the Ayahuasca Group in showing significantly higher scores on the mystical experience and near-death questionnaires and trends toward higher ratings of the personally meaning and spiritual significance of the experience as well as more positive life changes in social relationships, mood, and behavior. As noted in a previous survey [21] the similarities between the DMT and Ayahuasca groups despite differences in demographics and likely differences in the route of administration and context of use suggests that *N*,*N*- dimethyltryptamine produces a unique profile of effects that is phenomenologically distinct from two widely used classic psychedelics (psilocybin and LSD), which were indistinguishable on all measures assessed in this survey.

The present study builds upon previous observations by Timmermann et al. who characterized effects of intravenous DMT vs. placebo in drug experienced participants [38]. First, Timmermann et al. showed that DMT produced significant increases in the Greyson NDE scale with mean total score about 50% of maximum possible score. Participants in the present survey provided retrospective ratings on the NDE scale of a similar magnitude (about 50% of maximum) of experiences occasioned by vaporized/inhaled DMT, as well as by psilocybin, LSD and ayahuasca. Second, Timmermann et al. showed a very high correlation between NDE Scale and MEQ ratings. We also observed a strong, significant positive association between the NDE Scale and the MEQ scale, within both the Non-Drug and Psychedelic Group. Finally, Timmermann et al. showed that the DMT-induced increases in total NDE scores were of similar magnitude to those observed in a small group (*n* = 13) of selectively recruited individuals who rated non-drug near-death experiences 7 years after their experiences. The present study extends these findings by showing that DMT, psilocybin, LSD, and ayahuasca all were associated with significantly higher ratings on the NDE Scale relative to the large group of respondents who responded based on a non-drug near-death or other non-ordinary experience that changed attitudes about death. However, in the present study, there was considerable variability within the Non-Drug group with respect to imminent danger and whether the experience was considered to be a near-death or other non-ordinary experience. Thus, it seems likely there may be subsets of persons with near-death experiences who would report NDE Scale ratings as high or higher as those whose experience was occasioned by psychedelics. Notably, the low prevalence of imminent danger in the Psychedelic group combined with the higher ratings on the NDE Scale in the Psychedelic Group suggest that the phenomenological characteristics of a near-death experience can occur outside of the context of threat of death.

### Strengths and limitations

Strengths of the present study include the examination of transformative experiences in a large sample using common measures that facilitate direct quantitative comparisons between psychedelic and non-drug experiences as well as between different psychedelic substances. Study limitations include the reliance on retrospective self-report to describe the transformation in death attitudes and the phenomenological characteristics of the experiences. Respondents were a self-selected study population that may not be representative of all psychedelic or near-death experiences. The study sample was primarily male, White, Non-Hispanic, and residing in the United States. Generalizability may be compromised given the low survey completion rate. The cross-sectional nature of the study prohibits strong inferences regarding causality, and differences between groups may have preceded the experience. Although statistical comparisons between groups controlled for several basic demographic characteristics that distinguished psychedelic and non-drug survey respondents including age, sex, education and income, additional factors such as ceremonial versus recreational setting were not assessed. Also, the survey was limited to a sub-set of classic psychedelic compounds and investigation of substances that share similar phenomenological characteristics (e.g., 5-MeO-DMT; ketamine) would be of interest. Despite our original intention to recruit primarily persons for the non-drug survey who reported having a near-death experience that transformed their perspective on dying, we observed a high percentage of non-drug survey respondents whose life was not in imminent danger or who self-identified as having an “other non-ordinary experience” vs. a “near-death experience.” The fact that participants’ personal characterization of near-death experiences differed from our anticipated conceptualization of near-death experiences suggests that more detailed measurement of the context of non-drug experiences that transform perspectives on death and dying is warranted. This could be accomplished in future research through specific follow-up questions or qualitative interviews that allow greater precision in distinguishing between different experiences (e.g., near-death experiences, out-of-body experiences, or experiences based on the death of a close family member).

## Conclusion

This study presents a detailed description and direct comparison of psychedelic drug-occasioned and non-drug experiences that changed perspectives on death and dying. Overall, the psychedelic and non-drug experiences showed striking similarities both in the phenomenological features of the experience as well as on changes attributed to the experience which included decreases in fear of death, positive changes in attitudes about death, and increases in personal well-being and life purpose and meaning. Although both psychedelic and non-drug participants showed robust increases on standardized measures of mystical and near-death experiences, these measures were significantly greater in the psychedelic participants. However, the non-drug participants were significantly more likely to rate their experiences as the single most meaningful, spiritually significant, insightful, and challenging of their lives. Comparing across psychedelic substances, ayahuasca and DMT groups tended to report stronger and more positive enduring consequences of the experience than the psilocybin and LSD groups, which were largely indistinguishable. Overall, the present findings, which show that both psychedelic and non-drug-occasioned experiences can produce positive and enduring changes in attitudes about death, suggest the importance of future prospective experimental and clinical observational studies to better understand mechanisms of such changes as well as their potential clinical utility in ameliorating suffering related to fear to death.

## Supporting information

S1 File (DOCX)
