# Supplementary material for: Comparison of psychedelic and near-death or other non-ordinary experiences in changing attitudes about death and dying
Source: PLoS One. 2022 Aug 24;17(8):e0271926. doi: 10.1371/journal.pone.0271926 (PMC9401141; doi:10.1371/journal.pone.0271926)
Supplement: S1 File — (DOCX) [file pone.0271926.s001.docx]

Supporting Information

Comparison of psychedelic and near-death or other non-ordinary experiences in changing attitudes about death and dying

Mary M. Sweeney^1^*, Sandeep Nayak^1^, Ethan S. Hurwitz^1,#a^, Lisa N. Mitchell^1^, T. Cody Swift^1,#b,#c^, Roland R. Griffiths^1,2^*

^1^ Johns Hopkins University School of Medicine, Department of Psychiatry and Behavioral Sciences, Behavioral Pharmacology Research Unit, Center for Psychedelics and Consciousness Research, Baltimore, Maryland, United States of America

^2^ Johns Hopkins University School of Medicine, Department of Neurosciences, Baltimore, Maryland, United States of America

^#a^ University of California, San Diego, Department of Psychology, San Diego, California, United States of America

^#b^ Current Address: Heffter Research Institute, Santa Fe, New Mexico, United States of America

^#c^ Current Address: RiverStyx Foundation, Santa Cruz, California, United States of America

* **Corresponding author(s):**

Mary M. Sweeney

Email: [msween19@alumni.jh.edu](mailto:msween19@alumni.jh.edu)

Roland R. Griffiths

Email: [rgriff@jhmi.edu](mailto:rgriff@jhmi.edu)

**S1 Table. Religious orientation and afterlife beliefs among the Psychedelic and Non-Drug groups**

|  | Non-Drug | Psychedelic |
| --- | --- | --- |
|  | n = 933 | n = 2259 |
| Beliefs in God |  |  |
| Percent endorsing belief about God’s existence |  |  |
| Believes God exists | 77% | 41% |
| Does not believe God Exists | 8% | 29% |
| Unsure | 14% | 31% |
| Religious orientation |  |  |
| Percent endorsing current orientation (non-mutually exclusive, in order of overall prevalence) | | |
| Christian | 34% | 11% |
| Agnostic | 8% | 28% |
| Pantheist (belief in the divinity of the universe) | 11% | 22% |
| Buddhist | 9% | 21% |
| Atheist | 3% | 16% |
| Nature-based | 7% | 19% |
| Earth-based | 6% | 14% |
| Secular (no religious or spiritual orientation) | 7% | 12% |
| Taoism | 4% | 9% |
| Pagan | 4% | 6% |
| Percent endorsing current orientation (mutually exclusive) |  |  |
| Atheist only | 1% | 7% |
| Any major monotheistic tradition only (Christian, Jewish, or Muslim) | 31% | 6% |
| Other (not atheist only or major monotheistic tradition only) | 68% | 87% |
| Beliefs in the afterlife |  |  |
| Percent endorsing belief about the afterlife (mutually exclusive) |  |  |
| There is some form of continuance after death other than the physical body |  |  |
| Yes | 90% | 58% |
| Possibly | 9% | 32% |
| No, absolutely not | 2% | 10% |
| Percent endorsing belief about the afterlife (non-mutually exclusive) |  |  |
| What do you believe happens after you die? |  |  |
| 1. “Nothing, I will cease to exist.” | 5% | 27% |
| 1. “I will go to a positive realm (e.g., heaven, nirvana, paradise, etc.)” | 51% | 21% |
| 1. “I will go to a negative realm (e.g., hell, a realm of suffering, etc.)” | 2% | 3% |
| 1. “I will go to a positive realm (e.g., heaven, nirvana, paradise, etc.) or a negative realm (e.g., hell, a realm of suffering, etc.)” | 11% | 12% |
| 1. “I will go to a neutral realm, separate from the conventional positive and negative realms” | 19% | 33% |
| 1. “I will be reincarnated” | 39% | 36% |
| 1. “There is a continuity of some form of consciousness” | 72% | 65% |
| 1. “There is a continuity of spirit” | 70% | 49% |
| 1. “There is a continuity of energy” | 68% | 66% |
| 1. “There is a continuity of soul” | 72% | 49% |
| 1. “There is continuity through family and friends” | 42% | 29% |
| Belief in any continuity of life after death (endorsed any item 2-11 above) | 99% | 96% |

**S2 Table. Religious orientation and afterlife beliefs among the Non-Drug, Psilocybin, LSD, Ayahuasca, and DMT groups**

|  | Non-Drug | Psilocybin | LSD | Ayahuasca | DMT |
| --- | --- | --- | --- | --- | --- |
|  | *n* = 933 | *n* = 766 | *n =* 904 | *n =* 282 | *n =* 307 |
| Beliefs in God |  |  |  |  |  |
| Percent endorsing current belief about God’s existence |  |  |  |  |  |
| Believes God exists | 77% | 39% | 36% | 58% | 46% |
| Does not believe God Exists | 8% | 27% | 34% | 17% | 25% |
| Unsure | 14% | 34% | 30% | 25% | 29% |
| Religious orientation |  |  |  |  |  |
| Percent endorsing current orientation (non-mutually exclusive, in order of overall prevalence) | | | |  |  |
| Christian | 34% | 11% | 14% | 7% | 6% |
| Agnostic | 8% | 29% | 29% | 20% | 27% |
| Pantheist (belief in the divinity of the universe) | 11% | 23% | 17% | 28% | 26% |
| Buddhist | 9% | 23% | 20% | 20% | 21% |
| Atheist | 3% | 14% | 19% | 7% | 16% |
| Nature-based | 7% | 22% | 15% | 22% | 19% |
| Earth-based | 6% | 16% | 12% | 15% | 16% |
| Secular (no religious or spiritual orientation) | 7% | 13% | 12% | 10% | 13% |
| Taoism | 4% | 9% | 9% | 7% | 12% |
| Pagan | 4% | 8% | 5% | 8% | 7% |
| Percent endorsing current orientation (mutually exclusive) |  |  |  |  |  |
| Atheist only | 1% | 5% | 10% | 3% | 6% |
| Any major monotheistic tradition only (Christian, Jewish, or Muslim) | 31% | 7% | 7% | 5% | 4% |
| Other (not atheist only or major monotheistic tradition only) | 68% | 88% | 83% | 92% | 90% |
| Beliefs in the afterlife |  |  |  |  |  |
| Percent endorsing belief about the afterlife (mutually exclusive) | |  |  |  |  |
| There is some form of continuance after death other than the physical body | | |  |  |  |
| Yes | 90% | 56% | 51% | 78% | 66% |
| Possibly | 9% | 36% | 35% | 17% | 27% |
| No, absolutely not | 2% | 8% | 14% | 5% | 6% |

| **S2 Table. (Continued) Religious and afterlife beliefs among the Non-Drug, Psilocybin, LSD, Ayahuasca, and DMT groups** | | | | | | | | | |
| --- | --- | --- | --- | --- | --- | --- | --- | --- | --- |
|  | Non-Drug | | | Psilocybin | | LSD | | Ayahuasca | DMT |
|  | *n* = 933 | | | *n* = 766 | | *n =* 904 | | *n =* 282 | *n =* 307 |
| Beliefs in the afterlife (Continued) | |  |  | |  | |  | |  |
| Percent endorsing belief about the afterlife (non-mutually exclusive) | |  |  | |  | |  | |  |
| What do you believe happens after you die? | |  |  | |  | |  | |  |
| “Nothing, I will cease to exist.” | | 5% | 27% | | 32% | | 13% | | 25% |
| “I will go to a positive realm (e.g., heaven, nirvana, paradise, etc.)” | | 51% | 21% | | 18% | | 23% | | 23% |
| “I will go to a negative realm (e.g., hell, a realm of suffering, etc.)” | | 2% | 3% | | 3% | | 4% | | 3% |
| “I will go to a positive realm (e.g., heaven, nirvana, paradise, etc.) or a negative realm (e.g., hell, a realm of suffering, etc.)” | | 11% | 13% | | 13% | | 7% | | 11% |
| “I will go to a neutral realm, separate from the conventional positive and negative realms” | | 19% | 32% | | 32% | | 29% | | 40% |
| “I will be reincarnated” | | 39% | 34% | | 36% | | 39% | | 36% |
| “There is a continuity of some form of consciousness” | | 72% | 66% | | 58% | | 75% | | 74% |
| “There is a continuity of spirit” | | 70% | 50% | | 45% | | 59% | | 53% |
| “There is a continuity of energy” | | 68% | 68% | | 61% | | 68% | | 72% |
| “There is a continuity of soul” | | 72% | 49% | | 45% | | 55% | | 53% |
| “There is continuity through family and friends” | | 42% | 30% | | 30% | | 28% | | 27% |
| Belief in any continuity of life after death (endorsed any item 2-11 above) | | 99% | 97% | | 96% | | 97% | | 96% |
